# Metal-induced oxidative stress and human plasma protein oxidation after SARS-CoV-2 infection

**DOI:** 10.1038/s41598-023-29119-5

**Published:** 2023-02-10

**Authors:** Baikuntha Aryal, Joseph Tillotson, Kiwon Ok, Andrew T. Stoltzfus, Sarah L. J. Michel, V. Ashutosh Rao

**Affiliations:** 1 Laboratory of Applied Biochemistry, Division of Biotechnology Research and Review III, Office of Biotechnology Products, Office of Pharmaceutical Quality, Center for Drug Evaluation and Research, Food and Drug Administrations, Silver Spring, MD 20993 USA; 2 Department of Pharmaceutical Sciences, University of Maryland School of Pharmacy, Baltimore, MD 21201 USA; 3 Present Address: Pfizer Inc., Cambridge, MA USA

**Keywords:** Viral infection, Iron, Metalloproteins

## Abstract

Pathogenesis of COVID-19 by SARS-CoV-2 resulted in a global pandemic and public health emergency in 2020. Viral infection can induce oxidative stress through reactive oxygen species (ROS). Inflammation and environmental stress are major sources of oxidative stress after infection. Micronutrients such as iron, copper, zinc, and manganese play various roles in human tissues and their imbalance in blood can impact immune responses against pathogens including SARS CoV-2. We hypothesized that alteration of free metal ions during infection and metal-catalyzed oxidation plays a critical role towards pathogenesis after infection. We analyzed convalescent and hospitalized COVID-19 patient plasma using orthogonal analytical techniques to determine redox active metal concentrations, overall protein oxidation, oxidative modifications, and protein levels via proteomics to understand the consequences of metal-induced oxidative stress in COVID-19 plasma proteins. Metal analysis using ICP-MS showed significantly greater concentrations of copper in COVID-19 plasma compared to healthy controls. We demonstrate significantly greater total protein carbonylation, other oxidative modifications, and deamidation of plasma proteins in COVID-19 plasma compared to healthy controls. Proteomics analysis showed that levels of redox active proteins including hemoglobulin were elevated in COVID-19 plasma. Molecular modeling concurred with potential interactions between iron binding proteins and SARS CoV-2 surface proteins. Overall, increased levels of redox active metals and protein oxidation indicate that oxidative stress-induced protein oxidation in COVID-19 may be a consequence of the interactions of SARS-CoV-2 proteins with host cell metal binding proteins resulting in altered cellular homeostasis.

## Introduction

Coronavirus disease 2019 (COVID-19) has infected more than 664 million people worldwide and has already claimed more than 6.7 million lives globally as of January 10, 2023. Several studies have been published since the widespread occurrence of severe acute respiratory syndrome coronavirus -2 (SARS-CoV-2); however, the exact pathogenesis of COVID-19 is not clearly understood. Dysregulation of innate and adaptive immune systems, cytokine storm, hypoxia, and thrombosis are considered key factors associated with the severity of COVID-19 disease^1^.

Upon inhalation, SARS-CoV-2 enters the host respiratory cells via interactions with its receptor, an angiotensin-converting enzyme 2 (ACE2), where it undergoes replication causing various inflammatory and immune reactions. While the detection of SARS-CoV-2 viral load in the respiratory tract is commonly used for the diagnosis of COVID-19, several reports indicate that SARS-CoV-2 RNAs are not always confined to the respiratory track but may also spread to other parts of the body including plasma and serum^2–4^. The detection of SARS-CoV-2 mRNA was highly correlated to the severity of COVID-19 symptoms in these reports. SARS-CoV-2 RNA was detected in the plasma of 27% of hospitalized COVID-19 patients and was correlated with respiratory disease severity, low lymphocyte counts, and increased inflammatory markers^3^.

Oxidative stress caused by an imbalance between reactive oxygen species (ROS) and antioxidants plays an important role for several pathological conditions including cardiovascular, neurodegenerative, cancer, and lung diseases. Accumulation of ROS can cause disruption of redox signaling and impart irreversible damage to lipids, proteins, and nucleic acids. ROS-induced oxidative stress causes posttranslational modifications of several amino acid residues in proteins such as carbonylation, dihydroxyphenylalanine (DOPA) formation, methionine oxidation, cysteine oxidation, and deamidation which may lead to structural and functional alteration of proteins^5^. While methionine or cysteine oxidation are reversible modifications, protein carbonylation is an irreversible posttranslational modification mostly detected in proline, arginine, lysine, and threonine residues. Most well-developed and commonly used methods to determine carbonylated proteins rely on the derivatization on the carbonyl moiety of amino acid residues with 2,4-dinitrophenylhydrazine (DNPH) resulting in the formation of a stable dinitrophenylhydrazone (DNP) complex with the protein. DNP-derivatized carbonylated proteins are then detected by ELISA, western blot immunoassays, or by mass spectrometry^6,7^.

Oxidative stress and inflammation have been linked to the pathogenesis of COVID-19, but the exact mechanism is not clearly understood^8,9^. Besides inflammation, several factors including hypoxia, endothelial dysfunction, iron metabolism dysregulation, and the release of free and labile iron are attributed to oxidative stress during the development of COVID-19 disease^10,11^. Several macro and micronutrients such as K, Ca, Mg, Fe, Cu, Zn, and Mn play important roles in host cell metabolic processes and the overall immune system including inflammatory responses and antiviral activity^12^. Therefore, measurement of metal concentrations in plasma of COVID-19 patients is hypothesized to be critical for understanding the severity of COVID-19. Metal-catalyzed oxidative stress is one of the mechanisms that involves activity of redox-active metal ions such as iron and copper to produce ROS. Metals are considered essential components of several viral proteins and play a key role in their survival and pathogenesis^13^. Molecular modeling performed to compare biological roles of SARS-CoV-2 proteins has found that viral proteins may interact with several human metalloproteins, and certain viral proteins could coordinate to react with heme on the 1-beta chain of hemoglobin of host cells to dissociate iron^14,15^. This could lead to increase in free metal concentration and oxidative stress due to production of ROS through metal-catalyzed redox process.

In this study, we analyzed COVID-19 convalescent plasma, plasma from symptomatic hospitalized COVID-19 patients, and plasma from healthy subjects using relevant advanced analytical techniques to understand oxidative stress and to identify potential drivers of pathogenesis in COVID-19.

## Materials and methods

### Human plasma samples

Convalescent COVID-19 plasma samples from adult donors recovered form COVID-19 and acute plasma samples from hospitalized COVID-19 adult patients were obtained from Washington Adventist Hospital, Maryland and Adventist Health Care White Oak Medical Center, Maryland respectively. Acute and convalescent COVID-19 plasma samples were collected between 4/21/2020 and 5/18/2020 and were heat inactivated in the hospital labs prior to arrival at the FDA. Informed consent was obtained from all participants. All samples were deidentified and the age, gender, other clinical conditions, and comorbidity factors associated with the patients were not disclosed. Plasma samples from healthy adult donors were purchased from SeraCare Life Sciences (Gaithersburg, MD). Healthy plasma samples were heat inactivated before use. A total number of 20 normal control, 10 acute COVID-19 and 30 convalescent plasma samples were used in our study. This research was approved by FDA’s Research Involving Human Subjects Committee (RIHSC 2020-04-02) and by the University of Maryland Baltimore Institutional Review Board, in accordance with the Committee’s requirements and the Declaration of Helsinki.

### Determination of protein carbonylation

Plasma protein concentration was determined using BCA assay. Quantification of protein carbonylation was performed using a modified ELISA method as described in a previous publication by our group^7^. Briefly, 10 µL of 1 µg/µL plasma proteins from each sample were denatured with 10 µL of 10% (w/v) sodium dodecyl sulfate (SDS) and derivatized with 20 µL of 20 mM 2,4-dinitrophenylhydrazine (DNPH) solution prepared in 10% (v/v) trifluoroacetic acid (TFA). Samples were incubated at room temperature for 10 min and the derivatization reaction was stopped with 20 µL of 2 M Tris base. An aliquot of 3 µL of each DNP-derivatized sample was diluted with 0.25 mL of adsorption buffer [20 mM NaHCO_3_, 150 mM NaCl, 0.25% SDS (w/v), pH 8.5], and 100 µL of diluted samples were loaded in duplicate into a 96-well Maxisorp plate. The plate was covered with aluminium foil and incubated overnight at 4 °C. After incubation, the sample plate was rinsed gently 6 times with PBST (1X PBS containing 0.05% Tween 20) and incubated with 200 µL per well of blocking buffer (1% BSA in PBST) for 1 h at 37 °C. The blocking buffer was discarded and incubated with 100 µL per well of blocking buffer containing goat anti-DNP antibody for 1 h at room temperature. Following incubation, the sample plate was rinsed 6 times with PBST, and incubated with horseradish peroxidase (HRP)-conjugated rabbit anti-goat IgG antibody for 1 h at room temperature. After washing 6 times with PBST, the plate was incubated with 100 µL per well of TMB substrate at room temperature for 2–3 min for color development. The reaction was stopped with 100 µL of 0.5 M H_2_SO_4_, and the absorbance was measured at 450 nm and 690 nm. After subtraction of background absorbance at 690 nm, the carbonyl content in each sample was determined using a standard curve generated from an oxidized BSA standard.

### Determination of protein carbonylation by western blot

Protein carbonylation was determined by derivatization of protein carbonyls with DNPH using a procedure based on previous publications^5^. Approximately 12 µg of plasma proteins were treated with 6% (w/v) SDS in a 30 µL volume. An equal volume of 20 mM DNPH in 10% (v/v) TFA was added and incubated at room temperature for 10 min. The reaction was neutralized with 30 µL of 2 M Tris in 30% (v/v) glycerol containing 7% (v/v) β-mercaptoethanol, and 15 µL of each DNP-derivatized sample was loaded in two identical gels. One gel was used for Coomassie staining, and proteins from other gel were transferred to an immobilon-P PVDF membrane (Millipore, Billerica, MA). After transfer, the PVDF membrane was immunoblotted with goat anti-DNP primary antibody (Bethyl Laboratories Inc., Montgomery, TX) and donkey anti-goat IRDye 800CW secondary antibody (LI-COR, Lincoln, NE). The DNP-derivatized carbonylated proteins were detected using the Odyssey infrared imaging system (LI-COR, Lincoln, NE).

### Determination of DOPA in plasma samples

DOPA formation was detected using a fluorescence-based detection method as previously described^16^. Plasma samples (40 µg each) were diluted with 300 µL of 100 mM sodium phosphate pH 9.2 and treated with 300 µL of 20 mM 4-(aminomethyl)-benzenesulfonic acid (ABS) prepared in 100 mM sodium phosphate pH 9.2. Each sample was then incubated with 6 µL of 100 mM potassium hexacyanoferrate(III) solution for 90 min in the dark at room temperature. DOPA standard solutions (0, 0.625, 1.25, 2.5, 5, and 10 µM) were prepared following the same procedure to generate a standard curve. 200 µL each of the DOPA standard and plasma samples were transferred to black 96 well flat-bottom plates in triplicate and the fluorescence intensity of each sample was measured by exciting at 360 nm and recording the emission spectrum at 690 nm using a SpectraMax3 micro plate reader (Molecular Devices).

### Protein identification

Protein bands of interest in 1D and 2D gels were excised and digested with sequencing grade trypsin/Lys-C mix (Promega Corporation, Madison, WI) for 16 h at 37 °C per the manufacturer’s instructions. The digested peptides were analyzed by Q-TOF LC/MS (Waters Corporation, Milford. MA). Peptide search and protein identification was performed using ProteinLynx Global Server (PLGS).

### 2D gel electrophoresis

DNPH derivation of plasma proteins and sample preparation for 2D gel electrophoresis was performed using a previously published method^17^. After transfer, the PVDF membrane was incubated with two primary antibodies for 3 h at room temperature, a goat polyclonal anti-DNP antibody (Bethyl Laboratories, Montgomery, TX) and anti-complement C3 (Cell Signaling, Danvers, MA). After three PBST washes, the membrane was incubated for 1 h at room temperature with donkey anti-goat 800CW (green) secondary antibody for carbonyl and donkey anti-rabbit 680LT (red) secondary antibody for complement C3. The membrane was then scanned with the Odyssey infrared imaging system (LI-COR Biosciences, Lincoln, NE) to visualize complement C3 and carbonylated protein spots.

### ICP-MS analysis

Plasma samples were prepared for Inductively Coupled Plasma Mass Spectrometry (ICP-MS) analysis by first adding concentrated nitric acid (60–70% w/w, TraceMetal Grade, Fisher Chemical) to plasma samples. The samples were then placed in an oven set to 80 °C for 4 h to allow for digestion, after which the samples were diluted with Chelex-treated Milli-Q water (Chelex 100 resin from Sigma, Elga LabWater PURELAB Milli-Q water system) to a final concentration of 6% HNO_3_, and again digested in the oven overnight at 80 °C. The samples were prepared in triplicate and analyzed the following day by ICP-MS. Atomic absorption standards (Fluka Analytical) for ^24^Mg, ^31^P, ^39^K, ^44^Ca, ^56^Fe, ^63^Cu, ^66^Zn were used to prepare a calibration curve of each of the elements at a concentration range of 2 to 2000 ppb in the same matrix as the samples (6% HNO_3_). An Agilent 7700 × ICP-MS instrument (Agilent Technologies, Santa Clara, CA, USA) with an Octopole Reaction System (ORS), in He_2_ mode, was used to detect these metals. Additional ICP-MS parameters include an RF power of 1550 W, an octopole RF of 190 V, an OctP bias of − 18 V, an argon carrier gas flow of 0.99 L/min, and a helium gas flow of 4.3 mL/min. Samples were directly infused via autosampler into the 7700 × peristaltic pump with a pump speed of 0.1 rps and a micromist nebulizer. Agilent’s Mass Hunter software was used for data extraction and quantitation of elements (ppb) based on the calibration curve and corrected for dilution.

### Mass spectrometry analysis

Plasma proteins (50 mg) were resuspended in 10% TCA in acetone, kept at − 20 °C for 90 min and centrifuged at 15,000×*g* for 10 min at 4 °C. Supernatant was discarded and the pellet was washed with 1 mL of cold 10%TCA/acetone and centrifuged at 15,000×*g* for 10 min at 4 °C. The pellet was air-dried and dissolved in resuspension buffer (8 M Urea, 50 mM Ammonium bicarbonate, 25 mM DTT) at 37 °C for 30 min. Reduced samples were treated with 50 mM iodoacetamide and incubated in the dark at room temperature for 30 min. Reduced and alkylated samples were diluted with 50 mM ammonium bicarbonate to reduce the urea concentration to less than 1 M. Samples were treated with trypsin at a trypsin to protein ratio of 1:100, incubated at 37 °C overnight, and purified using ZipTip following the Pierce C18 tips (Thermo, 87782) protocol.

Samples were analyzed using Waters Xevo G2-XS Q-TOF nano-LC/MS system, utilizing nano-Ease M/Z HSS C18 T3 column (100A, 1.8 mm, 75 mm × 100 mm)). A gradient of acetonitrile containing 0.1% formic acid (2–40% for 90 min, 40–90% for 91 min–95 min, 90–2% from 100 min–120 min) was used at a flow rate of 500 nL/min. Acquisition time started at 0 min and ended at 120 min in positive ion mode, under sensitivity analyzer. Mass range of 100 Da to 2000 Da with a threshold of 20,000 Da was used with a scan time of 0.2 s, continuum. Maximum ions selected for MS/MS was 3 and a collision energy ram that ranged from 8 to 15 V for LM CE and 50 V to 60 V HM CE.

### Identification of post translational modifications (PTM) of proteins/peptides

PEAKS Studio X + (Bioinformatics Solutions Inc.) was used to search the MS/MS spectra against the SwissProt protein database in human sequences. Parameters that were set are as follows: precursor tolerance = 10 ppm, product ion tolerance = 0.5 Da, carbamidomethylation was used as a fixed modification, and carbonylation, deamidation, DOPA, oxidation and double oxidation were used for variable modifications with a maximum missed cleavage of 2 and a 1% false discovery rate (FDR).

### Molecular modeling

#### Protein–ligand interactions

Molecular docking studies were conducted using PyMOL and AutoDock 4.2.6 to determine the binding mode of heme (with and without iron) with SARS-CoV-2 proteins. The structures of Nsp1, papain-like protease of Nsp3, ADP ribose protease of Nsp3, ubiquitin-like domain 1 of Nsp3, Nsp9, Nsp10-Nsp16 complex, Orf7a, main protease, and S protein receptor-binding domain were retrieved from the Protein Data Bank (PDB; PDB ID’s: 7K3N, 6W9C, 6WEN, 7KAG, 6W4B, 6YZ1, 6W37, 6Y2E, and 6W41, respectively). The envelope protein, M protein, and Orf10 are proposed models derived from SWISS-MODEL based on target amino-acid sequences and formatted as a PDB file. Structures were prepared using a receptor preparation script in AutoDockTools by deleting water, if any, adding hydrogens, and assigning partial charges (compute gasteiger). Protoporphyrin IX (CID: 4971) was downloaded in SDF format and was prepared using a ligand preparation script in AutoDockTools. The receptor and ligand were docked using default parameters with 25 number of runs. The conformations were chosen based on the lowest binding energy.

#### Protein–Protein interactions

The HDOCK Server (hust.edu.cn) was used to predict interactions between hemoglobin (PDBID: 1A3N) and the envelope protein, m protein, or Orf10 (all proposed models). In short, the PDB file for hemoglobin was uploaded as the receptor molecule and the viral proteins were uploaded as the ligand molecule.

### Statistical analyses

Statistical analyses were performed with the GraphPad Prism (GraphPad; La Jolla, CA). As indicated in the figure legends, a two-way ANOVA or unpaired two tailed t-test, where appropriate, were used to determine significant differences. *p < 0.05, **p < 0.001, ***p < 0.0001.

## Results

### Total plasma protein carbonylation increases in COVID-19 plasma samples

**Figure 1:**
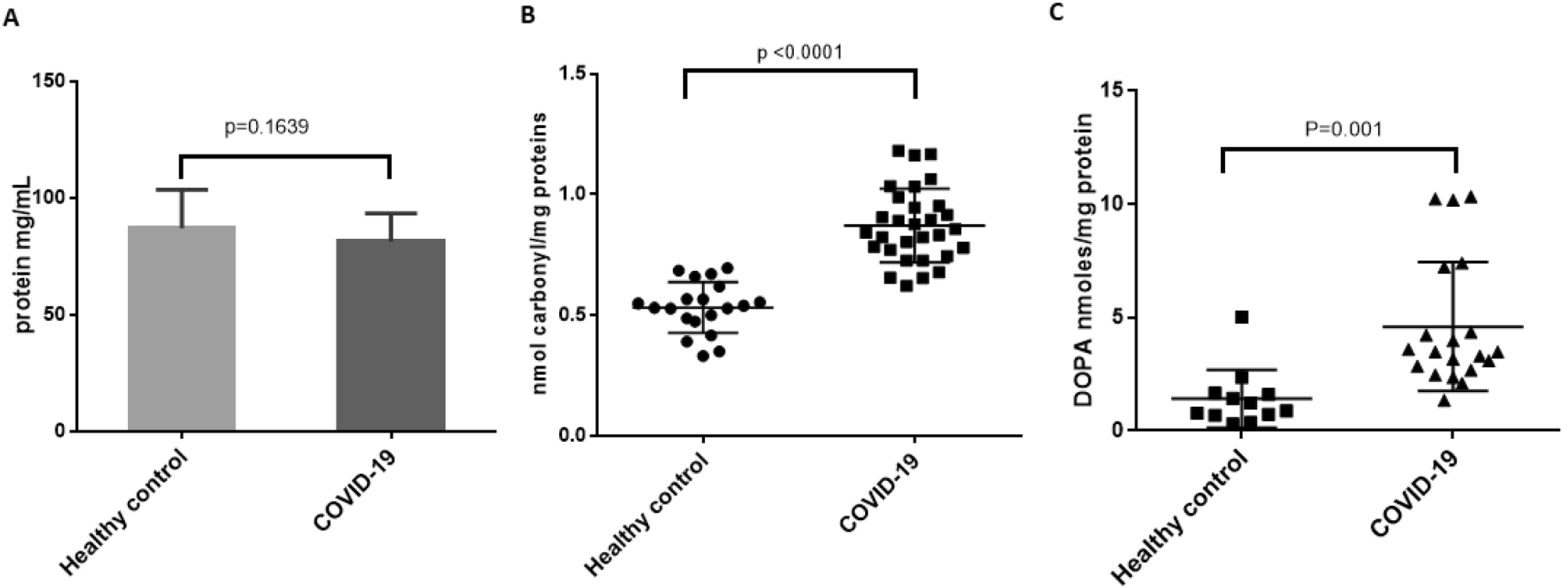
Total plasma protein and oxidative stress-induced plasma protein oxidation. (**A**) total plasma protein concentrations determined by BCA, (**B**) Total plasma protein carbonylation and (**C**) Total DOPA formation in plasma proteins. Each closed circle (●), triangle (▲) or square (■) represents the average value determined from at least three independent measurements for each sample. COVID-19 samples in panels A-C include both convalescent and acute COVID-19 plasma samples. For individual results see Supplementary Figs. S3, S4 and S5. Statistical significance was determined using GraphPad Prism using unpaired t-test.

We first determined the total protein concentration in healthy controls as well as acute and convalescent COVID-19 plasma samples. Our results showed no significant changes in the total plasma protein concentration in COVID-19 samples compared to plasma samples from healthy controls (Fig. 1A). We then determined the total protein carbonylation in plasma samples using a quantitative ELISA assay. Our results showed that total plasma protein carbonylation was significantly increased in both convalescent COVID-19 plasma samples and acute COVID-19 plasma samples compared to healthy controls (Fig. 1B). There was no significant difference in the total protein carbonylation between COVID-19 convalescent plasma samples and plasma samples from the acute, hospitalized COVID-19 patients (Figs. S3 and S4). Therefore, we grouped all acute and convalescent COVID-19 plasma samples together as COVID-19 samples and compared with the normal controls. We calculated total protein carbonylation as nmol/mg plasma protein (Fig. 1B) and nmol/mL plasma (not shown) and in both cases total protein carbonylation was significantly higher in the COVID-19 plasma compared to that of the healthy controls. Since the exact ages of the patients for the deidentified COVID-19 plasma samples used in our study were not disclosed by the hospital and based on their protocol only adults were involved in this study population; we used normal control plasma samples with ages ranging from 22 to 71 years old to address any potential age-related differences in total protein carbonylation. We compared total protein carbonylation in normal control plasma samples separately between an age group of less than 40 years and an age group greater than 40 years. Our data shows that there was no significant difference in total protein carbonylation between these two age groups (Fig. S5).

### Total 3,4 dihydroxyphenylalanine (DOPA) increases in COVID-19 plasma samples

Tyrosine residues in proteins are susceptible to oxidation under oxidative stress conditions that may impact protein quality. Tyrosine oxidation to 3,4 dihydroxyphenylalanine (DOPA) has been previously reported to be a common modification under oxidative stress conditions^16,18^. We used a fluorescence-based method to measure DOPA levels in the plasma samples. The acute and convalescent COVID-19 plasma samples showed a significant increase in DOPA formation compared to the healthy control samples (Fig. 1C). Consistent with total protein carbonylation, there was no significant difference in DOPA formation between COVID-19 convalescent plasma samples and plasma samples from the acute COVID-19 patients (Fig. S6).

### Plasma protein levels of selected proteins are altered in COVID-19 plasma compared to healthy plasma samples

**Figure 2:**
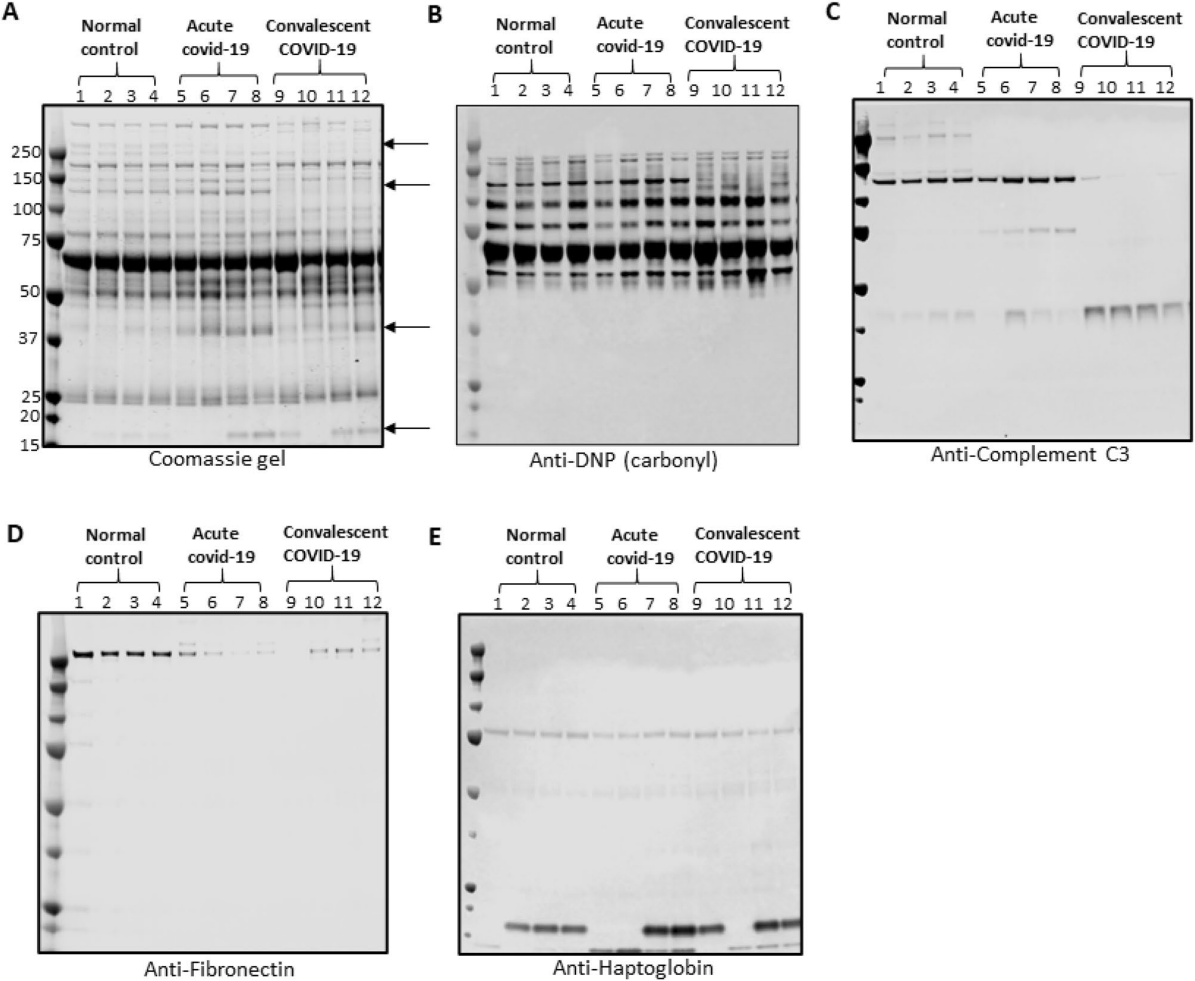
Plasma protein analysis by SDS-PAGE. (**A**) representative Coomassie stained SDS-PAGE gel, (**B**) representative western blot using anti-DNP antibody for carbonylated proteins, (**C**) representative western blot for complement C3 (**D**) representative western blot for fibronectin and (**E**) representative western blot for haptoglobin. Arrows in panel A represent specific proteins detected at different levels in normal control, acute COVID-19, and convalescent plasma.

To identify specific proteins that are impacted by oxidative stress and differential expression of such proteins due to SARS-CoV-2 infection, plasma samples were analyzed by SDS-PAGE with Coomassie staining or western blotting using antibodies that are specific to proteins of interest identified by mass spectrometry. Visualization of Coomassie stained gel showed distinct differences in ~ 260 kDa, ~ 115 kDa, ~ 42 kDa, and 16 kDa bands between healthy and COVID-19 samples (Figs. 2A, S1). Using mass spectrometry, these specific protein bands were identified to be fibronectin (~ 260 kDa) complement C3 (~ 115 kDa and ~ 42 kDa bands) and haptoglobin (~ 16 kDa band). Identity of these proteins was further confirmed using antibodies against each identified protein (Fig. 2A,C–E). The ~ 115 kDa complement C3 band was predominantly present in healthy plasma samples and acute COVID-19 plasma samples but not in convalescent COVID-19 plasma samples. Similarly, the ~ 42 kDa band of complement C3 was predominantly present in convalescent COVID-19 samples with only faint bands present in acute COVID-19 and healthy plasma samples. Consistent with bands on the Coomassie stained gel, the level of haptoglobin was greater in COVID-19 samples compared to healthy but the 16 kDa haptoglobin band was missing in some samples. Western blot analysis for protein carbonylation showed carbonylation of complement C3 (115 kDa band) in healthy and acute COVID-19 plasma samples but carbonylation was not observed for the same protein band in the convalescent COVID-19 plasma samples (Fig. 2B).

### 2D gel electrophoresis confirms the degradation of Complement C3 in COVID-19 plasma samples

To further investigate specific protein carbonylation and to confirm degradation of complement C3 in the plasma samples, we performed 2D gel electrophoresis. Two-colored western blot analysis (red for complement C3 and green for carbonyl) showed only fragments of complement C3 in the convalescent COVID-19 samples but both full-length complement C3 and fragments were observed in the healthy control plasma samples. Two carbonylated protein spots (Fig. S8, spots a and b) that were distinct from the healthy plasma samples show partial overlap with complement C3 fragments. While other high molecular weight proteins show some degree of differences in carbonylation between healthy and COVID-19 samples with high carbonylation in COVID-19 compared to healthy plasma samples, these two spots were consistently observed in all five randomly selected convalescent COVID-19 samples for 2D gel electrophoresis but were absent in the healthy control plasma samples. Mass spectrometry analysis of protein corresponding to these two carbonylated spots identified as complement C3 fragments. Based on peptide coverage, the high molecular weight fragment of the carbonylated spot (Fig. S8, spot a) was identified as C-terminal fragment of complement C3 covering amino acid residues from 1321 to 1663 and the low molecular weight fragment of the carbonylated spot (Fig. S8, spot b) was identified as complement C3 fragment covering amino acid residues from 981 to 1260.

### Proteomic analysis confirms greater posttranslational modifications in COVID-19 plasma samples

**Figure 3:**
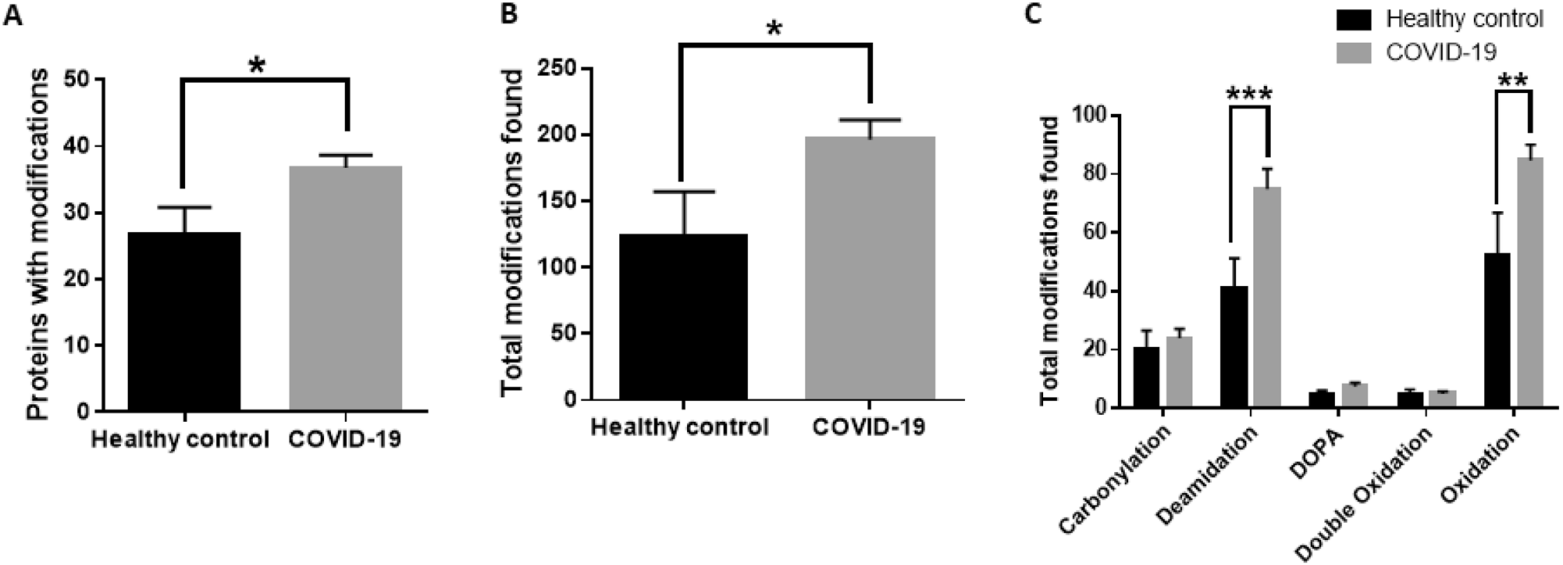
Post-translational modifications associated with oxidation and/or deamidation in healthy control and COVID-19 samples. (**A**) The total number of proteins with post-translational modifications analyzed in this study. (**B**) The total number of amino acid modifications in total plasma proteins. (**C**) The total number of amino acid modifications found in total plasma proteins based on specific modifications. COVID-19 samples in panels (**A**–**C**) include both convalescent and acute COVID-19 samples. Statistical analysis was performed using GraphPad Prism using unpaired t-test for (**A**) and (**B**) and a two-way ANOVA for (**C**). (*p < 0.05; **p < 0.01; ***p < 0.001).

**Figure 4:**
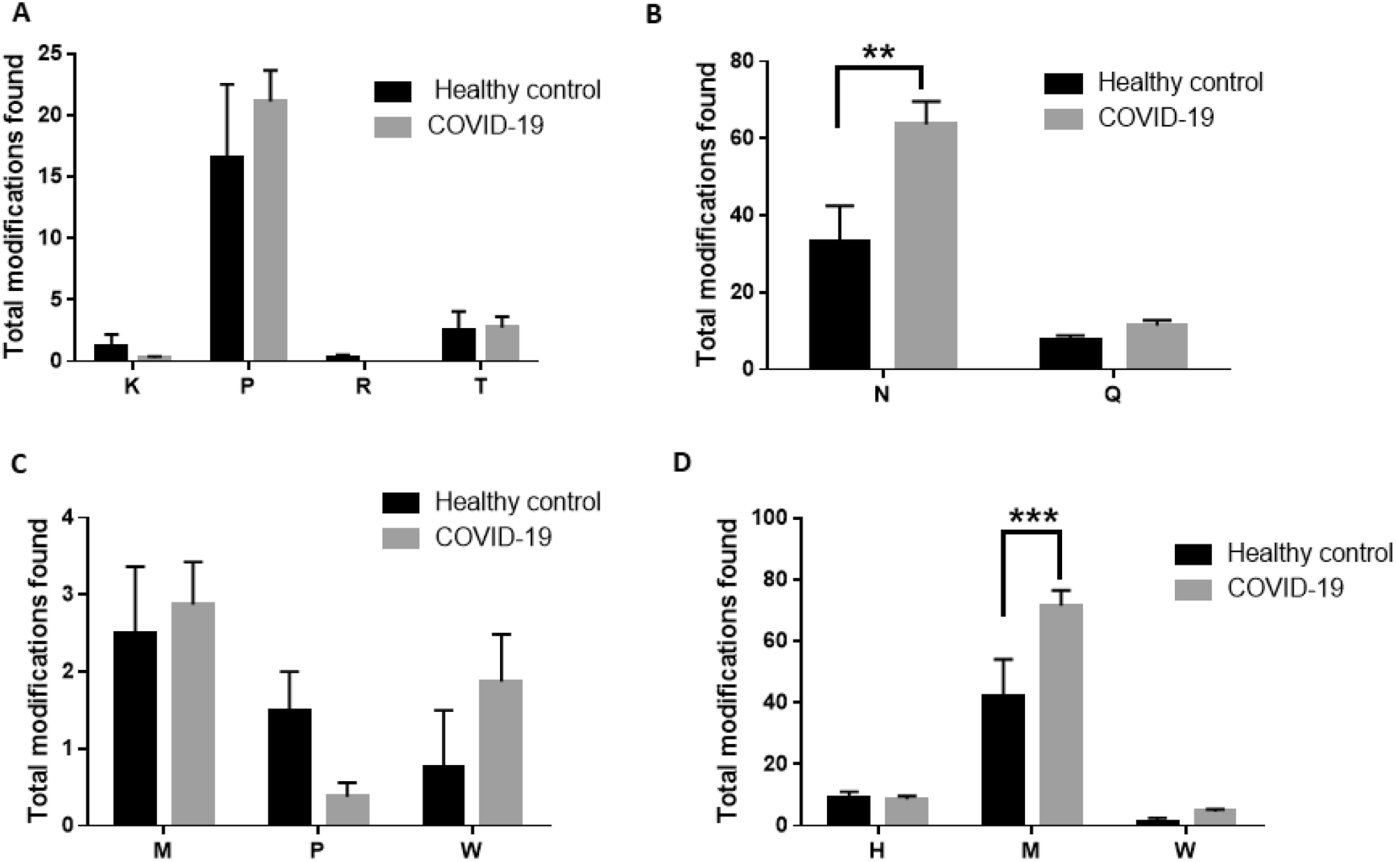
Specific post-translational modifications in healthy control and COVID-19 samples. The total number of amino acid modifications in total plasma proteins based on (**A**) carbonylation, (**B**) deamidation, (**C**) double oxidation, and (**D**) oxidation. Statistical analysis was performed using GraphPad Prism using two-way ANOVA. (*p < 0.05; **p < 0.01; ***p < 0.001). COVID-19 samples in panels (**A**–**D**) include both convalescent and acute COVID-19 samples.

Mass spectrometry data of plasma samples were analyzed by PEAKS Studio X + software using the SwissProt protein database to understand post-translational modifications in plasma proteins after SARS CoV-2 infection. We observed a significantly greater number of proteins with post-translational modifications (carbonylation, oxidation, and deamidation) and a significantly greater total number of post-translational modifications in COVID-19 plasma samples compared to healthy control plasma samples (Fig. 3A, 3B, Table 1). Specifically, the total number of deamidation and oxidized sites were significantly higher in COVID-19 plasma samples compared to healthy controls (Fig. 3C). To further understand amino acids residues that are susceptible to deamidation or oxidation during SARS-CoV-2 infection, we analyzed the total number of modifications in each amino acid residue. Asparagine (N) was found to be the primary deamidation site and methionine (M) was found to be the primary oxidation sites for post-translationally modified proteins (Fig. 4A–D). We then analyzed protein expression level in COVID-19 and control plasma samples and reported major proteins that show higher than threefold changes in expression level. We observed 7 different proteins including a heme-binding protein that showed greater than threefold increase in expression level in COVID-19 plasma samples compared to healthy controls (Table 2).

**Table 1:** Proteomic analysis of plasma samples from healthy controls and COVID-19 patients for post-translational modifications.

Protein ID	Proteins	Healthy control	COVID-19	Protein ID	Proteins	Healthy control	COVID-19
P01024|CO3_HUMAN	Complement C3	23	19	P19652|A1AG2_HUMAN	Alpha-1-acid glycoprotein 2	3	5
P01023|A2MG_HUMAN	Alpha-2-macroglobulin	10	20	Q14624|ITIH4_HUMAN	Inter-alpha-trypsin inhibitor heavy chain H4	1	7
P02787|TRFE_HUMAN	Serotransferrin	21	26	A0A0B4J1X5|HV374_HUMAN	Immunoglobulin heavy variable 3–74	1	1
P0DOX5|IGG1_HUMAN	Immunoglobulin gamma-1 heavy chain	33	34	P01042|KNG1_HUMAN	Kininogen-1	1	2
P02768|ALBU_HUMAN	Albumin	6	24	P08603|CFAH_HUMAN	Complement factor H	4	3
P02675|FIBB_HUMAN	Fibrinogen beta chain	16	19	P05155|IC1_HUMAN	Plasma protease C1 inhibitor	1	6
P01859|IGHG2_HUMAN	Immunoglobulin heavy constant gamma 2	29	24	P01008|ANT3_HUMAN	Antithrombin-III	1	1
P01861|IGHG4_HUMAN	Immunoglobulin heavy constant gamma 4	16	20	P06312|KV401_HUMAN	Immunoglobulin kappa variable 4–1	1	1
P01876|IGHA1_HUMAN	Immunoglobulin heavy constant alpha 1	6	7	P04430|KV116_HUMAN	Immunoglobulin kappa variable 1–16	1	0
P00738|HPT_HUMAN	Haptoglobin	17	30	P01624|KV315_HUMAN	Immunoglobulin kappa variable 3–15	1	1
P01860|IGHG3_HUMAN	Immunoglobulin heavy constant gamma 3	15	19	A0A075B6S5|KV127_HUMAN	Immunoglobulin kappa variable 1–27	0	1
P01009|A1AT_HUMAN	Alpha-1-antitrypsin	7	11	P04114|APOB_HUMAN	Apolipoprotein B-100	1	11
P0DOX7|IGK_HUMAN	Immunoglobulin kappa light chain	9	6	P00450|CERU_HUMAN	Ceruloplasmin	0	9
P01834|IGKC_HUMAN	Immunoglobulin kappa constant	8	5	P01011|AACT_HUMAN	Alpha-1-antichymotrypsin	0	7
P02671|FIBA_HUMAN	Fibrinogen alpha chain	10	12	P02647|APOA1_HUMAN	Apolipoprotein A-I	0	2
P0C0L4|CO4A/B_HUMAN	Complement C4-A	3	6	P01019|ANGT_HUMAN	Angiotensinogen	0	1
P00739|HPTR_HUMAN	Haptoglobin-related protein	10	14	P68871|HBB_HUMAN	Hemoglobin subunit beta	0	2
P02679|FIBG_HUMAN	Fibrinogen gamma chain	9	18	P04003|C4BPA_HUMAN	C4b-binding protein alpha chain	0	1
P02790|HEMO_HUMAN	Hemopexin	9	9	P04004|VTNC_HUMAN	Vitronectin	0	2
P01871|IGHM_HUMAN	Immunoglobulin heavy constant mu	2	4	P19827|ITIH1_HUMAN	Inter-alpha-trypsin inhibitor heavy chain H1	0	1
P0DOY2|IGLC2_HUMAN	Immunoglobulin lambda constant 2	1	1	A0A0B4J1X5|HV374_HUMAN	Immunoglobulin heavy variable 3–74	0	1
P02765|FETUA_HUMAN	Alpha-2-HS-glycoprotein	6	5	P00747|PLMN_HUMAN	Plasminogen	0	1
P0DOX2|IGA2_HUMAN	Immunoglobulin alpha-2 heavy chain	3	4	P69905|HBA_HUMAN	Hemoglobin subunit alpha	0	1
P02763|A1AG1_HUMAN	Alpha-1-acid glycoprotein 1	2	6	P0DP09|KV113_HUMAN	Immunoglobulin kappa variable 1–13	0	1
P04217|A1BG_HUMAN	Alpha-1B-glycoprotein	1	1	A0A0C4DH72|KV106_HUMAN	Immunoglobulin kappa variable 1–6	0	1
P02749|APOH_HUMAN	Beta-2-glycoprotein 1	2	3	P02750|A2GL_HUMAN	Leucine-rich alpha-2-glycoprotein	0	1
P01780|HV307_HUMAN	Immunoglobulin heavy variable 3–7	1	1	P02753|RET4_HUMAN	Retinol-binding protein 4	0	1
P10909|CLUS_HUMAN	Clusterin	1	1				

The listed proteins were selected based on having at least one post-translational modifications such as oxidation, carbonylation, and/or deamidation. Numbers in healthy control and COVID-19 columns indicate number of post-translational modifications for the corresponding protein.

**Table 2:** Expression of proteins in plasma samples of COVID-19 patients compared to healthy controls.

Accession	Peptides	Score	ANOVA (p)	Fold change	Protein description
P00738	89 (82)	190.78	0.02	3.49	Haptoglobin
P68871	23 (21)	190.55	0.02	5.06	Hemoglobulin subunit beta
P01011	18 (18)	184.59	2.50e−0.003	3.65	Alpha-1-antichymotrypsin
P0DJ18	3 (1)	43.17	0.08	40.68	Serum amyloid A-1 protein
P02741	3(3)	24.12	0.01	14.20	C-reactive protein
P69905	10 (10)	96.59	7.46e−0.003	9.17	Hemoglobin subunit alpha
P0DJ19	5 (3)	64.97	0.06	10.86	Serum amyloid A-2 protein

Proteins with higher than threefold changes in expression level are listed in the Table.

### Molecular docking of SARS-CoV2 proteins with porphyrin and hemoglobin

To understand the interaction of viral proteins with iron binding proteins in the host cell, molecular docking studies were performed to determine potential protein–ligand interactions between selected viral proteins and protoporphyrin IX, with and without iron (Table 3). Our analysis for calculation of the theoretical binding energy (kcal/mol) shows that several viral proteins may have the potential to interact with a porphyrin group with or without bound iron. The envelope protein has the highest docking affinity (most negative value of binding energy) and Orf10 showed the second highest interaction with porphyrin regardless of bound iron. The M protein had the third highest docking affinity to porphyrin without iron while the ubiquitin-like domain of Nsp3 showed the third highest interaction to porphyrin with iron. Interestingly, spike protein with most positive binding energy indicates minimal or no interaction with porphyrin regardless of bound iron.

**Table 3:** Theoretical binding energies of respective SARS-CoV-2 proteins with porphyrin with or without iron.

Protein	Porphyrin w/o Fe (kcal/mol)	Porphyrin w/ Fe (kcal/mol)
Envelope (monomer)	− 8.42	− 8.96
Envelope (pentamer)	− 13.96	− 12.42
M protein	− 9.35	− 10.23
Nsp1	+ 706.88	+ 684.32
Nsp3 (ADP phosphatase)	+ 81.9	+ 123.5
Nsp3 (protease)	+ 72.7	+ 130.5
Nsp3 (ubiquitin-like domain)	− 8.7	− 10.5
Nsp9	+ 2.71	+ 14.15
Nsp10	− 4.93	− 6.76
Nsp16	+ 594.77	+ 563.52
Orf7a	− 9.14	− 9.23
Orf10	− 12.76	− 11.76
Protease	+ 149.59	+ 13.52
Spike (RBD)	+ 900.32	+ 824.25

The possibility for these viral proteins to interact with porphyrin rings led us to investigate whether similar binding relationships can be seen with heme-containing proteins, specifically hemoglobin. The three proteins that had the best calculated binding energy with porphyrin (envelope protein, m protein, and Orf10) were docked with hemoglobin using the HDOCK server (Table 4). The interactions proposed in our study were based on the best docking energy score obtained from the molecular modeling.

**Table 4:** Docking studies of respective SARS-CoV-2 proteins with deoxyhemoglobin bound to heme.

Viral protein	Docking score	Ligand RMSD (Å)	Model
Envelope (pentamer)	− 251.28	52.71	
M protein	− 316.5	66.3	
Orf10	− 278.32	26.79	

Docking was performed using the HDOCK server. Magenta color represents α-chain of deoxyhemoglobin, green color represents β-chain of deoxyhemoglobin, and cyan color represent viral protein.

### ICP-MS shows Mg, Cu and K are significantly altered in COVID-19 plasma samples compared to healthy controls

**Figure 5:**
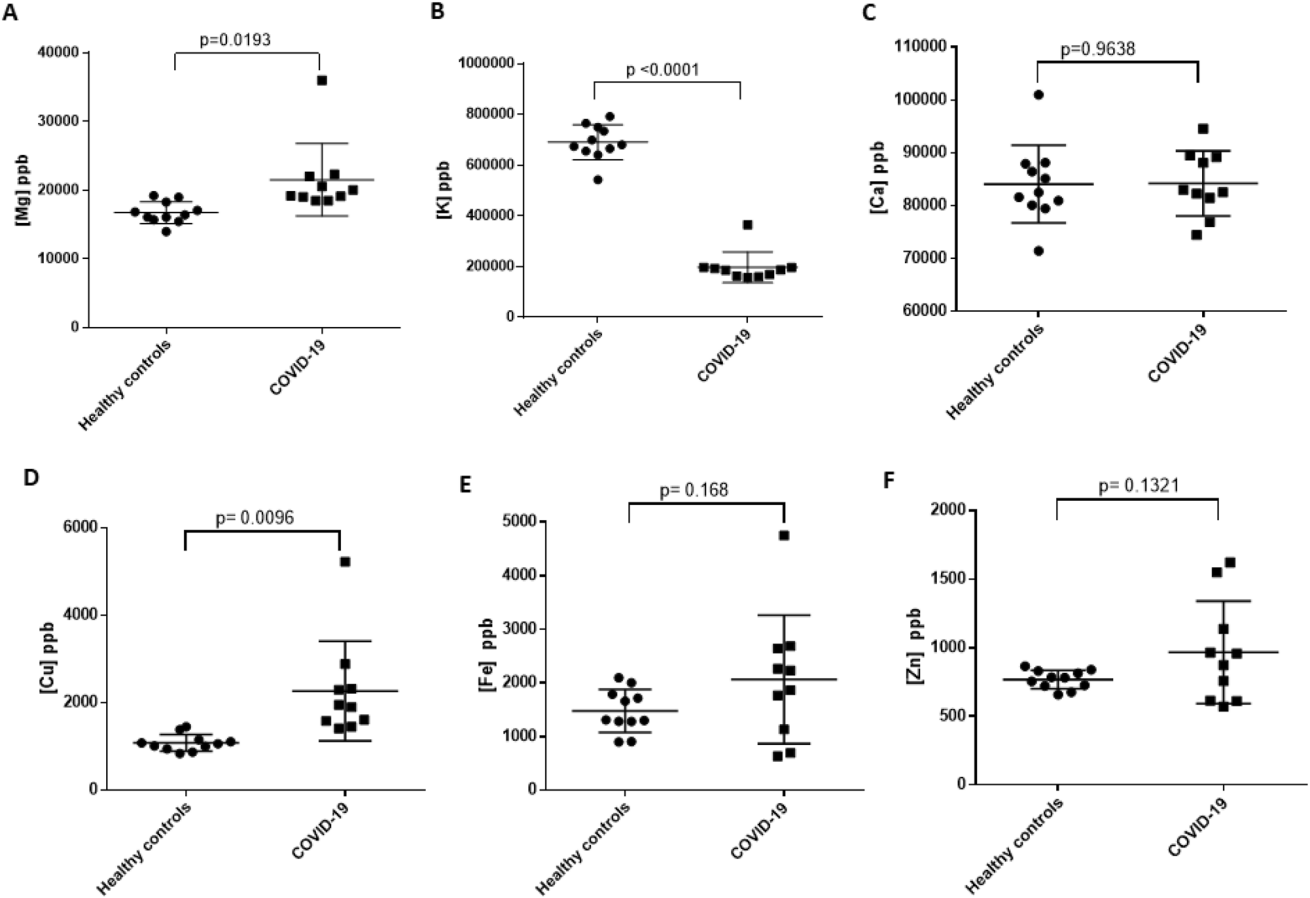
ICP-MS analyses of COVID-19 and healthy control plasma samples for magnesium (**A**), potassium (**B**), calcium (**C**) copper (**D**), iron (**E**) and zinc (**F**). Each closed circle (●) or square (■) represent the average value determined from at least three independent measurements for each specific sample. COVID-19 samples in panels (**A**–**F**) represent both convalescent and acute COVID-19 samples. Statistical significance was determined using GraphPad Prism using unpaired t-test.

To further understand the involvement of metal ions in oxidative stress during SARS-CoV-2 infection, we analyzed total calcium (Ca), potassium (K), magnesium (Mg), iron (Fe), copper (Cu) and zinc (Zn) levels in plasma samples using ICP-MS. Our results show that Mg and Cu concentrations are significantly increased, and K concentration is significantly decreased in acute and convalescent COVID-19 plasma samples compared to healthy controls (Fig. 5). Similarly, Fe and Zn concentrations trended slightly higher in the acute and convalescent COVID-19 samples compared to healthy controls, but the differences were not statistically significant. We also observed relatively high variability in metal concentrations of Fe, Zn and Cu in acute and convalescent COVID-19 samples compared to healthy controls. Ages of patients for COVID-19 plasma samples used in our study were not disclosed by the hospital; therefore, to account for any age-related differences in total metal content, we compared the metal content in normal control plasma samples between samples grouped by age less than 40 years or greater than 40. Our ICP-MS analysis showed that there is no significant differences in the metal content between these two groups (Fig. S7) for all metals analyzed in our study.

## Discussion

We performed a comprehensive investigation of COVID-19 patient plasma samples using a wide range of advanced analytical techniques to identify oxidative stress and triggers of inflammation after SARS-CoV-2 infection. Our data indicates that the total plasma protein concentration was not changed after SARS-CoV-2 infection (Fig. 1A); however, proteomic analyses revealed significant changes in specific protein levels in both acute and convalescent COVID-19 plasma samples compared to healthy controls (Table 2). Plasma’s proteomic profile is known to alter during inflammation, aging, angiogenesis, and cancer development^19^. Changes in plasma protein profiles during COVID-19 infection have also been previously reported and the extent of differential expression was reported to be dependent on the severity of COVID-19^20,21^. Proteins identified in these studies were enriched in specific biological processes including cytokine signaling, Alzheimer’s disease, coronary artery disease, complement activation, neutrophil activation, and T cell suppression. Therefore, additional studies supporting the identification of specific blood biomarkers can help to define the factors associated with severity of disease and to predict clinical outcome to improve prognosis of COVID-19.

Protein carbonylation by oxidation is an irreversible process that marks proteins for proteasomal degradation and is considered a biologically relevant marker to determine oxidative stress. Lysine, proline, threonine, and arginine are known to be more susceptible to oxidative carbonylation after severe oxidative stress^5,22,23^. Our studies are the first to reveal a significant increase in total protein carbonylation and DOPA formation in acute and convalescent COVID-19 plasma samples compared to healthy controls indicative that SARS-CoV-2 infection increases oxidative stress in COVID-19 patients. Specific posttranslational modifications analyzed by mass spectrometry showed a greater number of proteins with oxidation, carbonylation and deamidation in COVID-19 samples compared to healthy control regardless of whether the plasma samples were collected from hospitalized symptomatic COVID-19 patients or convalescent plasma from patients recovering after SARS-CoV-2 infection (Table 1). Out of 53 proteins identified with oxidative modifications or deamidation, 38 proteins showed an increased level of oxidation and deamidation in COVID-19 samples compared to healthy control and 15 proteins showed either no change or higher level of oxidation or deamidation in healthy control. Our results indicate that oxidative stress increases after SARS-CoV-2 infection and some protein markers remain elevated in convalescent samples. Previous studies have shown major roles of proinflammatory cytokine storm in acute respiratory distress syndrome and COVID-19 pathogenesis. Indeed, some viruses alter the cellular redox balance and induce oxidative stress in host cells during early infection which is critical for virus entry and replication^24^. Virus-induced oxidative stress activates the innate immune system through cytokine release and NF-κB activation^25^. While several factors can influence oxidative stress during infection, our work was focused on metal-catalyzed oxidative stress and protein oxidation. Metal-catalyzed oxidation (MCO) reactions are commonly known to generate ROS and induce protein oxidation. Redox-active metals such as copper and iron have well established mechanisms of metal-catalyzed protein oxidation^22,26^. MCO induces both protein carbonylation and DOPA formation in recombinant proteins^16^. Therefore, we hypothesized that redox-active free metal ions in plasma play a critical role for elevating plasma protein carbonylation in COVID-19 plasma samples, as demonstrated by our study.

It has been shown that certain micronutrients such as Zn, Cu, Fe, and Se have important roles in immune and antiviral response. Dysregulation of serum metal ion concentrations can impact immune responses to viruses and it has been previously linked to the severity of COVID-19 but the exact causal relationship is unknown^12^. To establish metal content in plasma samples and identify potential roles of these metal ions in SARS-CoV-2 infection, we analyzed convalescent COVID-19, acute COVID-19, and healthy control plasma samples by ICP-MS. Our quantification of metals that can be redox active (i.e., copper and iron) demonstrated that the copper concentration increases significantly in COVID-19 plasma samples compared to healthy controls without significant changes in iron concentration. This results was unexpected, as the prediction was that iron levels would be affected by COVID-19, not copper^14^. This prediction was based upon theoretical studies that suggested dysregulation of iron during viral infection as well as some experimental data. Another theoretical study conducted using a structure-based modeling approach revealed that most human metalloproteins including Zn, Fe, Cu, Mg, and Mn binding proteins interact with the SARS-CoV-2 orf8 protein with Zn binding proteins being the main target because of their high abundance in humans^15^.

Serum copper and zinc levels have been reported to increase significantly in COVID-19 pregnant women compared to control group pregnant women with similar demographic characteristics; however, the exact cause of this change and biological consequences is not known^27^. Similarly, elevated levels of copper and ceruloplasmin, a copper transport protein, concentrations were found in surviving COVID-19 patients compared to healthy control or non-survivors indicating regulation of copper metabolism during COVID-19 infection^28^. An elevated level of copper was also correlated with COVID-19 severity^12^. Under normal conditions, copper levels are tightly controlled in cells to prevent toxicity. Within the cell, copper chaperones serve to deliver copper to copper binding metalloproteins, and metallothionein serves as a sink for excess copper to bind^29–32^. Consequently, “free” copper levels remain low preventing toxicity. When copper levels are elevated, the copper trafficking, chaperoning and storage functions can be overloaded leading to an increase in “free” copper. This has the potential to cause oxidative stress as copper can undergo redox chemistry to produce damaging reactive oxygen species. In fact, copper has been shown to be more potent than iron to induce metal-catalyzed oxidative damage to proteins^33^. Therefore, the increased copper concentration detected in COVID-19 plasma samples in our study could be one of the critical factors that drive irreversible oxidative damage to proteins.

Conserved domain analysis, homology modeling, and molecular docking has indicated that certain SARS-CoV-2 surface proteins attack the heme on hemoglobin to dissociate iron from porphyrin and suppress heme metabolism^14^. Concomitantly, increased serum iron concentrations have been reported in studies of metal levels after SARS-CoV-2 infection and it was predicted that this increase in iron was due to degradation of hemoglobin and iron release. The potential consequence of these events can result in oxidative stress and protein oxidation. Additionally, the E protein on the surface of SARS-CoV-2 can bind iron or heme and has the conserved domains of cytochrome C oxidase, Fe-SOD, catalase, and peroxidase. After heme binding, E protein can produce superoxide, hydrogen peroxide and hydroxyl radicals damaging the host cells or tissues exposed on the surface of viruses^34^. Based on our results, plasma iron concentration did not change after infection in the hospitalized patient samples or the samples from patients recovering from COVID-19. Previous studies have suggested a correlation between severity of COVID-19 disease and serum iron deficiency after infection^35,36^ but in our study, we were not able to test this correlation because the samples, obtained during early days of the COVID-19 pandemic, did not have detailed information on the severity of COVID-19 for each patient and the exact sampling time after infection. Significant increase in ferritin-bound iron concentration has been reported in COVID-19 plasma compared to its normal level and such increase in ferritin iron concentration correlated well with the severity of COVID-19 symptoms^37^. The measurement of iron concentrations in the form of ferritin may not accurately reflect the total iron concentration in serum because serum ferritin is an acute phase response protein, and it does not accurately correlate with the iron availability during pro-inflammatory conditions such as COVID-19.

Interestingly, we also observed a significant decrease in potassium levels and increase in magnesium levels in both acute and convalescent COVID-19 plasma samples compared to healthy control. Hypokalemia resulting from the degradation of angiotensin-converting enzyme 2 has been reported in COVID-19 and our results are consistent with the data reported in the previous publications^38^.

SDS-PAGE and mass spectrometric proteomic analysis confirmed a reduction in fibronectin, a protein involved in blood clotting, in COVID-19 plasma samples compared to healthy control possibly due to degradation during SARS-CoV-2 infection. Our data are consistent with the previous publications where a lower level of fibronectin has been reported in critically ill COVID-19 patients which is attributed to high blood clotting with disseminated intravascular coagulation and organ failure^39,40^.

SDS-PAGE and proteomic analysis also show fragmentation of the complement C3 protein specifically in convalescent COVID-19 plasma whereas only partial fragmentation was observed in acute COVID-19 patient plasma samples. Complement system is critical as a first line host defense against bacterial, virus or fungus infection and can be activated via three different pathways leading to proteolytic cleavage to form smaller and relatively stable fragments. The plasma concentration of active complement C3 factors (fragments) are generally measured to assess immune response to viral infection^41^. The disappearance of the main C3 band and increased level of C3 fragments in convalescent COVID-19 plasma indicates the activation of an innate immune response of these patients that is sustained during recovery. Our data indicates a partial activation of the complement system in hospitalized acute COVID-19 patients as indicated by the partial degradation of C3 main band in plasma samples of acuteCOVID-19 patients (Fig. 2C). Although some studies suggest the role of complement activation in the pathogenesis of SARS-CoV-2 infection by triggering a severe cytokine storm, the reported results on the role of the complement system in COVID-19 are contradictory^42–44^. Therefore, plasma levels of C3 alone may not be a reliable marker for predicting COVID-19 progression.

To understand if oxidative stress drives the fragmentation of complement C3, we performed two-color western blot analysis using antibodies against complement C3 and DNP, a compound used to selectively derivatize and detect carbonyl modification in proteins. We found carbonylated protein bands of around 50 kDa selectively in all convalescent COVID-19 plasma samples tested by 2D gel electrophoresis which was markedly absent in healthy control (Fig. S8). Mass spectrometry analysis of corresponding spots in Coomassie stained 2D gel confirmed that these are the C-terminal fragments of complement C3. Based on amino acid sequence coverage, these fragments correspond to C3c (amino acid sequence 1321–1663) and C3dg (amino acid sequence: 981–1260). We also observed a partial overlap between C3c fragment and carbonylation bands but could not detect C3dg fragment using an antibody against complement C3. It is possible that oxidation of this fragment may interfere with the antibody recognition and its detection in the western blot. Overall, these findings further support that oxidative stress may be, at least in part, driving the fragmentation of complement C3.

Molecular modeling studies were performed to compare the theoretical binding energies of protoporphyrin IX (with or without iron) to the selected SARS-CoV-2 proteins. The values obtained from AutoDock were compared to determine potential binding interactions between porphyrin and the viral proteins. We also utilized HDOCK to test the binding relationship between 3 viral proteins that had the best calculated binding energy and hemoglobin, a heme-containing protein. Although there is no empirical evidence to support these interactions, it provides a hypothesis generating idea that the interactions between SARS-CoV-2 proteins and metal-binding containing proteins influence free metal concentrations, whether it be from an increase in free metals resulting in metal-catalyzed protein oxidation or a decrease in metals available for key enzymatic reactions essential for maintaining cellular homeostasis.

It should be noted that our exploratory study was limited to a relatively small sample size (10 acute COVID-19, 30 COVID-19 convalescent, and 20 healthy plasma samples). We also acknowledge the limited clinical information that was available at the time these samples were obtained, during the height of the COVID-19 pandemic between April and May of 2020. Detailed demographics and other comorbidity factors would be important to incorporate into future, larger studies. Our study provides a comprehensive analysis of the plasma samples investigated to address metal-catalyzed and oxidative stress-induced protein oxidation in COVID-19 and novel experimental evidence that oxidative stress may be a consequence of the interactions of SARS-CoV-2 proteins with host cell metal binding proteins resulting in altered cellular homeostasis and reduced oxygen consumption.

## Conclusions

This is the first report demonstrating that total protein oxidation and copper concentrations are significantly elevated after SARS-CoV-2 infection regardless of whether plasma samples were collected from symptomatic hospitalized patients or from patients recovering after SARS-CoV-2 infection (convalescent plasma). We propose that the observed increase in plasma copper concentration after SARS-CoV-2 infection is critical in inducing oxidative stress and protein oxidation via carbonylation. The totality of our experimental findings and theoretical analysis supports a model by which viral proteins interact with the host cell’s metal binding proteins leading to a release of the metal co-factors. This, in turn, likely promotes oxidative stress and abrogates the biological activity of impacted metalloproteins. Additional studies with a larger number of samples taken at various stages of infection are required to further confirm this hypothesis and understand the kinetics and mechanism of metal ion release upon SARS-CoV-2 infection as well as the involvement of these metal ions to induce cellular events downstream of oxidative stress, inflammation, and pathogenesis after infection.

## Supplementary Information

Supplementary Figures.

## Data Availability

All data supporting the findings of this study are available within the paper and its supplementary information file. Access numbers to the datasets generated and or analyzed during the current study are available in the UniProt repository. [Accession Numbers: P01024, P01023, P02787, P0DOX5, P02768, P02675, P01859, P01861, P01876, P00738, P01860, P01009, P0DOX7, P01834, P02671, P0C0L4, P00739, P02679, P02790, P01871, P0DOY2, P02765, P0DOX2, P02763, P04217, P02749, P01780, P10909, P19652, Q14624, A0A0B4J1X5, P01042, P08603, P05155, P01008, P06312, P04430, P01624, A0A075B6S5, P04114, P00450, P01011, P02647, P01019, P68871, P04003, P04004, P19827, A0A0B4J1X5, P00747, P69905, P0DP09, A0A0C4DH72, P02750, P02753, P00738, P68871, P01011, P0DJI8, P02741, P69905, P0DJI9].
