# Supplementary material for: Metal-induced oxidative stress and human plasma protein oxidation after SARS-CoV-2 infection
Source: Sci Rep. 2023 Feb 10;13:2441. doi: 10.1038/s41598-023-29119-5 (PMC9916496; doi:10.1038/s41598-023-29119-5)
Supplement: Supplementary file 1 — Supplementary Figures. [file 41598_2023_29119_MOESM1_ESM.docx]

Supplementary information

**Metal-induced oxidative stress and human plasma protein oxidation after SARS-CoV-2 infection**

Baikuntha Aryal^1^, Joseph Tillotson^1,2^, Kiwon Ok^3^, Andrew T. Stoltzfus^3^, Sarah L. J. Michel^3^, V. Ashutosh Rao^1,*^

^1^Laboratory of Applied Biochemistry, Division of Biotechnology Research and Review III, Office of Biotechnology Products, Office of Pharmaceutical Quality, Center for Drug Evaluation and Research, Food and Drug Administrations, Silver Spring, Maryland, 20993, USA, ^2^Current affiliation: Pfizer Inc., ^3^Department of Pharmaceutical Sciences, University of Maryland School of Pharmacy, Baltimore, Maryland 21201

*Corresponding author: [ashutosh.rao@fda.hhs.gov](mailto:ashutosh.rao@fda.hhs.gov); phone: 240-402-7338

**Keywords:** COVID-19, SARS-Cov2, protein oxidation, metal binding, carbonylaton, oxidative stress

**Running title:** Metal-induced oxidative stress in human COVID-19 plasma


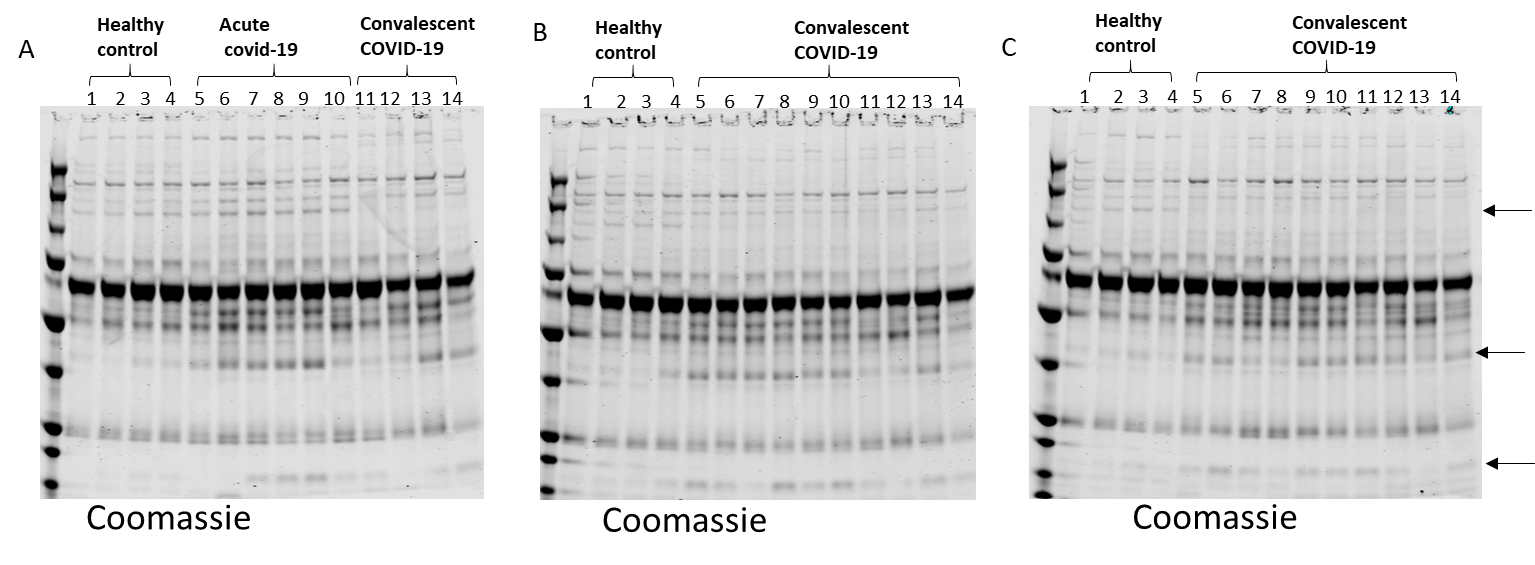


**Supplementary figure S1**: Comparison of protein levels in normal and COVID-19 plasma samples. 12 healthy control samples and 30 COVID-19 plasma samples were used in this study that includes 6 acute (gel # A, lanes 5-10) and 24 convalescent COVID-19 plasma samples.


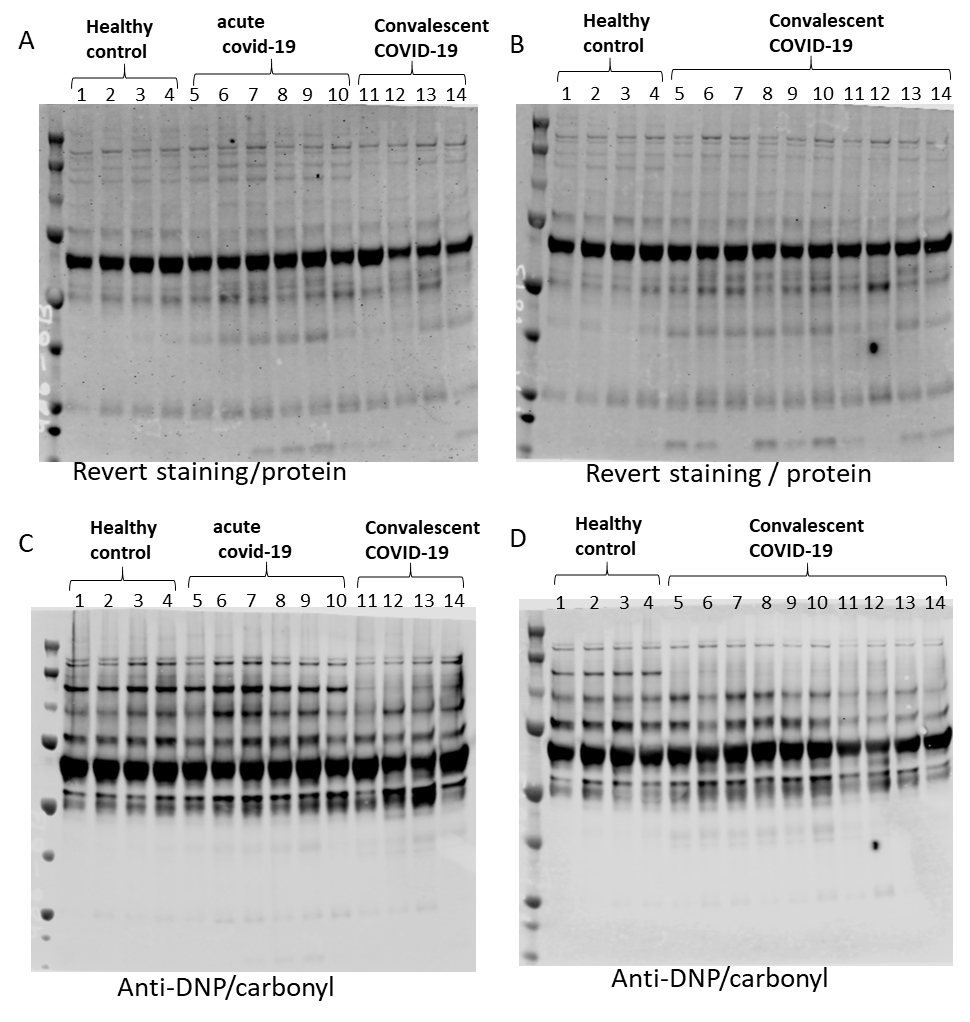


**Supplementary Figure S2**: Carbonylation of healthy control, acute infection, and convalescent COVID-19 plasma proteins. Plasma samples were loaded in two gels and plasma proteins were transferred to PVDF membrane. Total plasma proteins in membranes were first stained with revert staining (A and B), destained, and then incubated with anti-DNP antibody to detect carbonylated proteins in the same membranes (C and D).


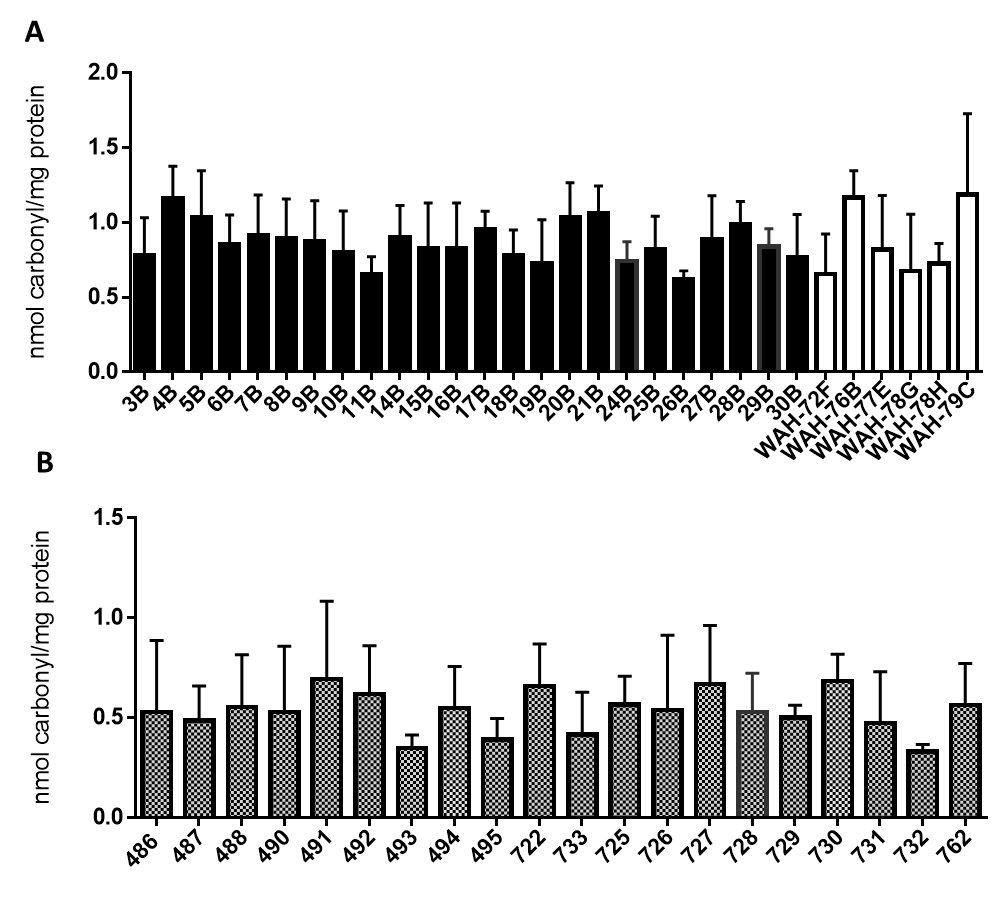


**Supplementary Figure S3**: Total carbonylation in COVID-19 (A) and healthy controls (B). Total protein carbonylation value for each sample was determined from three independent measurements. Samples WAH-72F, WAH-76B, WAH-77E, WAH-78G, 7WAH-8H, and WAH-79C (open bars on panel A) are acute COVID-19 plasma and all other samples (black bars on panel A) are COVID-19 convalescent plasma samples.


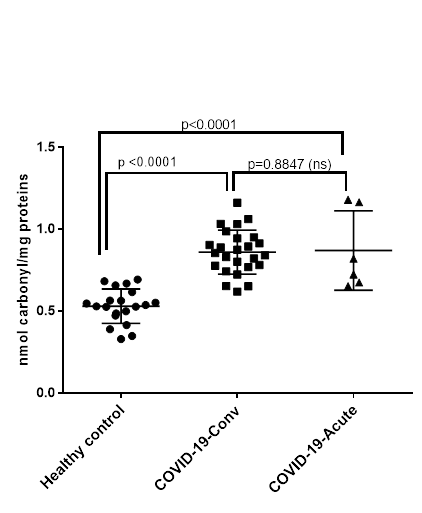


**Supplementary Figure S4**. Analysis of total protein carbonylation in healthy control, acute COVID-19, and convalescent COVID-19 plasma samples. All results from Figure S3 were used for statistical analysis to determine significant differences between healthy control vs acute COVID-19, healthy control vs convalescent COVID-19 plasma and acute COVID-19 vs convalescent COVID-19 plasma samples. Statistical analysis was performed using one-way-ANOVA.


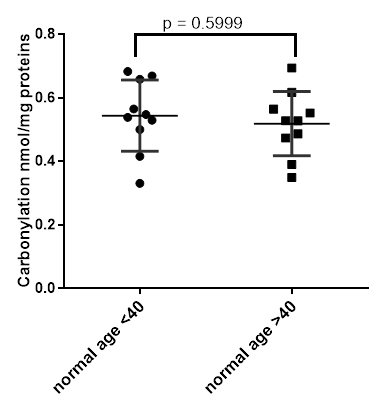


**Supplementary Figure S5**. Total protein carbonylation in normal control plasma samples. Carbonyl levels in normal control plasma samples with age group less than 40 (22-40 years) were compared with the age group greater than 40 (41-71 years) for total plasma protein carbonylation. Statistical analysis was performed using unpaired two tailed t-test.


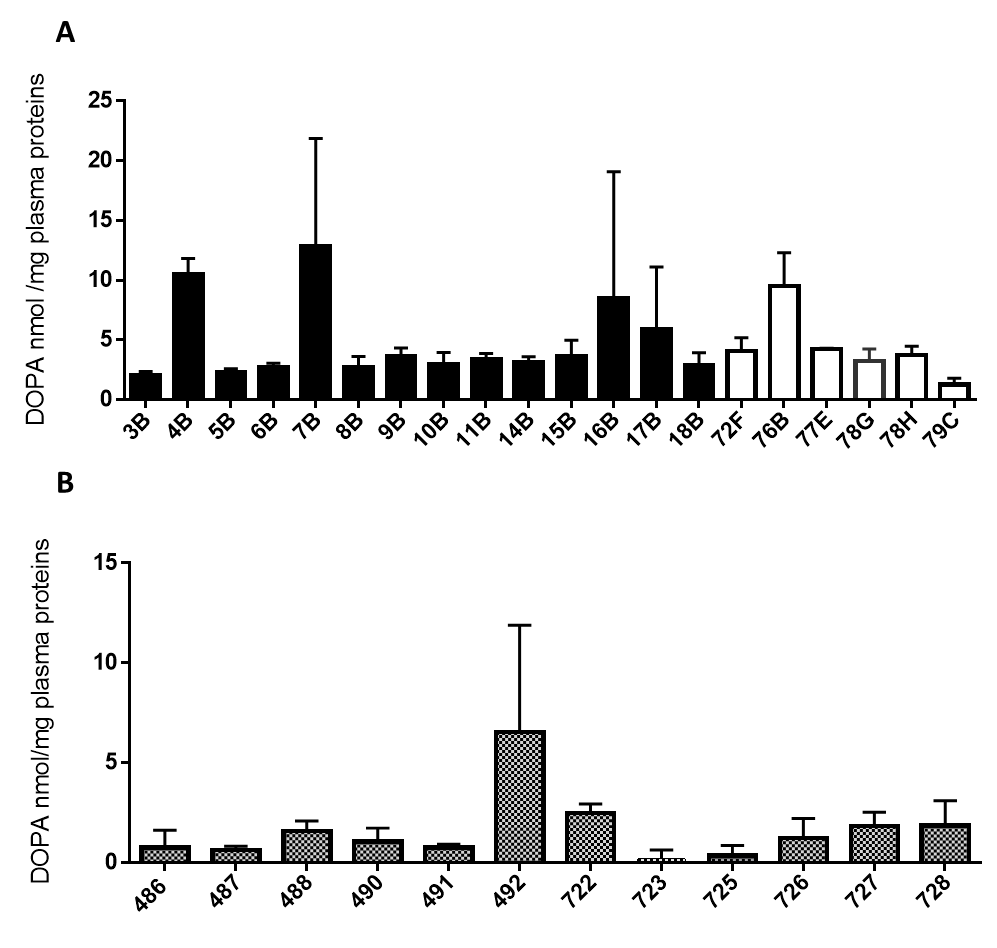


**Supplementary Figure S6:** Total DOPA formation in acute and convalescent COVID-19 plasma samples (A) and normal controls (B). DOPA was determined from three independent measurements. Sample # 72F, 76b, 77e, 78g, 78h, 78c (open bars in panel A) are acute COVID-19 plasma samples and all other samples (black bars in panel A) are COVID-19 convalescent plasma





**Supplementary Figure S7**. Metal analysis in normal control plasma samples. Metal content in normal control plasma samples used in our study with age group less than 40 (22-40 years) were compared with the age group greater than 40 (41-71 years) for Cu (A), Fe (B), Zn (C), K (D), Mg (E) and Ca (F). Statistical analysis was performed using unpaired two tailed t-test.


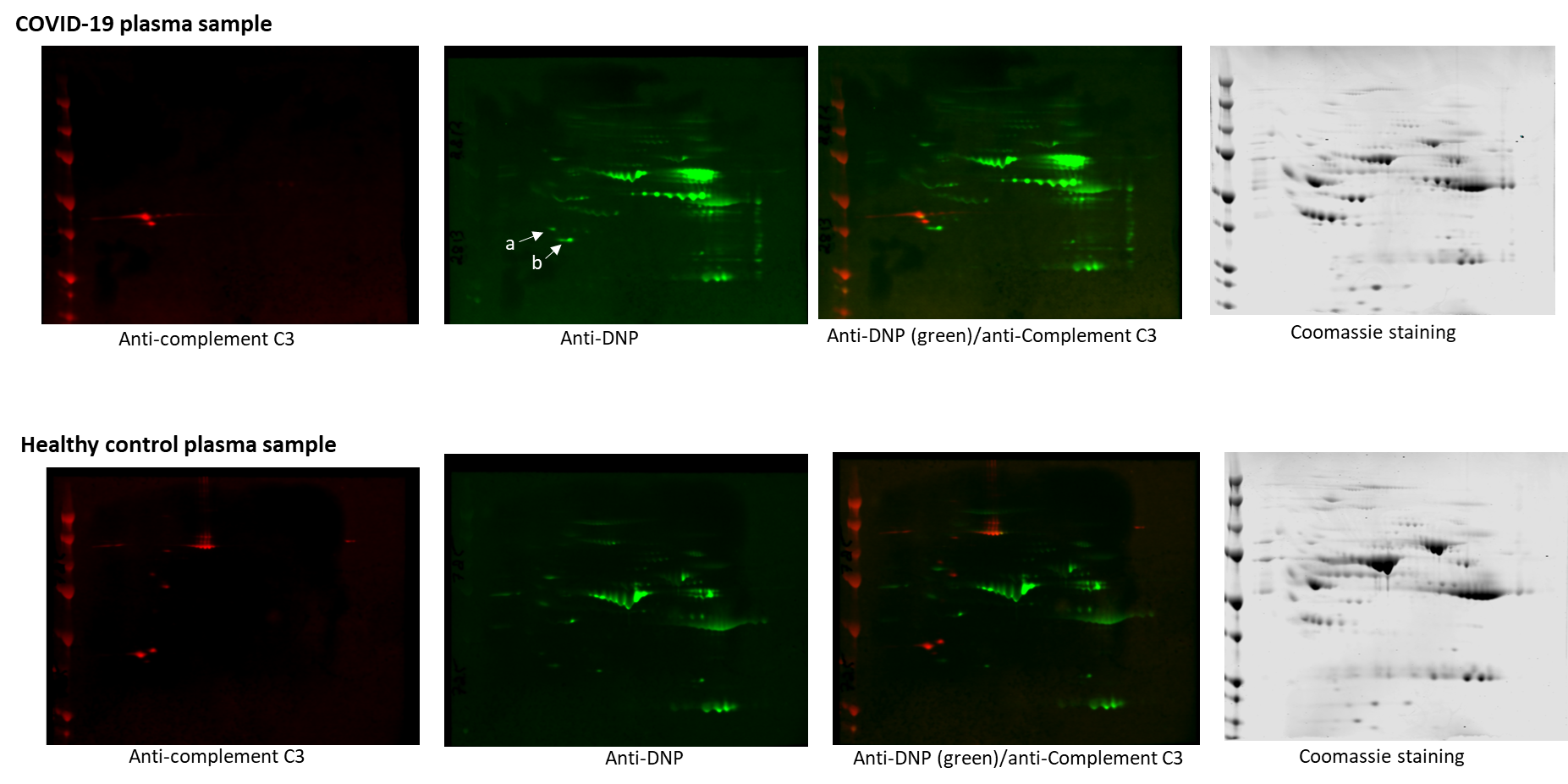


**Supplementary Figure S8**: Representative two-color 2D western for carbonyl and complement C3. Spots # a and b in anti-DNP western blot indicate specific carbonylated proteins in COVID-19 samples that are distinct from heathy control.
